# Molecular, Immunomodulatory, and Histopathological Role of Mesenchymal Stem Cells and Beetroot Extract on Cisplatin Induced Testicular Damage in Albino Rats

**DOI:** 10.3390/ani11041142

**Published:** 2021-04-16

**Authors:** Marwa T. Hassen, Hanaa K. Mohamed, Metwally M. Montaser, Mohamed E. El-Sharnouby, Nabil Awad, Rasha A. Ebiya

**Affiliations:** 1 Department of Zoology, Faculty of Women for Arts, Science and Education, Ain Shams University, Cairo 11757, Egypt; drhanaa_eta@yahoo.com (M.T.H.); meropinky87@gmail.com (H.K.M.); Rasha_Ali_511@yahoo.com (R.A.E.); 2 Science and Technology Department, University College of Ranyah, Taif University, Ranyah 21975, Saudi Arabia; 3 Department of Biotechnology, College of Science, Taif University, Taif 21944, Saudi Arabia; m.sharnouby@tu.edu.sa; 4 Department of Genetics, Faculty of Agriculture and Natural Resources, Aswan University, Aswan 81528, Egypt; nabil.faris@must.edu.eg; 5 College of Biotechnology, Misr University for Science and Technology, Giza 12563, Egypt

**Keywords:** cisplatin, testicular damage, caspase-3, StAR, iNOS, mesenchymal stem cells, beetroot extract

## Abstract

**Simple Summary:**

The chemotherapeutic agent Cisplatin (Cis) has testicular damage as a side effect. Therefore, efforts are being done by scientists to get over this effect. The current experiment was done to utilize bone marrow-derived stem cells (BM-MSCs) and beetroot extract (BRE) in reducing the Cis testicular damage in rats. In the current study, Cis reduced the sperm count, plasma testosterone level, the testicular activity of alkaline phosphatase beside a marked inhabitation of succinate dehydrogenase activity. Also, it significantly increased malondialdehyde and along with a marked decrease in testis reduced glutathione content and total antioxidant capacity. At the same time, Cis administration resulted in a marked elevation in interleukine-6 and the iNOS and caspase-3 genes, however it decreased the expression of steroidogenic acute regulatory protein (StAR). Stem cell therapy (BM-MSCs) was accompanied with the use of herbal therapy (BRE) resulted in great improvement of all previous parameters. These results were confirmed by histopathological and immunohistochemical examination. In conclusion the current study recommends the use of beetroot as natural food in combination with stem cell therapy for the patient suffering from the testicular side effect of cisplatin chemotherapy.

**Abstract:**

Cisplatin (Cis) a drug commonly used as a chemotherapeutic agent to treat various types of cancer, inducing testicular damage. The present study aimed to investigate the inhibitory potential of bone marrow-derived mesenchymal stem cells (BM-MSCs) and beetroot extract (BRE) in albino rats after testicular toxicity induced by cisplatin. Thirty adult male albino rats were grouped into: the control group, Cis group receiving a single dose of 7 mg/kg i.p. (intraperitoneal) to induce testicular toxicity, Cis plus BM-MSCs injected Cis followed by 2 × 10^6^ of BM-MSCs; Cis plus BRE group receiving Cis followed by 300 mg/kg body weight/day of BRE, and Cis plus BM-MSCs and BRE group. In the current study, Cis reduced sperm count, serum testosterone level, and testicular activity of alkaline phosphatase (AKP), besides a marked inhibition of succinate dehydrogenase (SDH) activity. In addition, it significantly increased malondialdehyde (MDA) and along with a marked decrease in testis reduced glutathione content and total antioxidant capacity (TAC). At the same time, Cis administration resulted in a marked elevation in interleukine-6 and the iNOS and caspase-3 genes; however, it decreased the expression of steroidogenic acute regulatory protein (StAR). Combined treatment with BM-MSCs and BRE resulted in great improvement of all previous parameters. These results were also confirmed by histopathological and immunohistochemical examination. In conclusion, both MSCs and BRE were found to have potent potentials to inhibit testicular damage induced by cisplatin.

## 1. Introduction

Infertility and its associated issues is a major problem faced by couples [1]. The most common reason for infertility in men is the inability to produce a sufficient number of active and healthy sperms [2]. Numerous aspects can affect sperm development, including the use of antibiotics, toxins, chemotherapy drugs for cancer, pesticides, air pollution, radiation, stress, and inadequate vitamins. Agarwal et al. [3] found that the causes noted decrease sperm levels by creating free radicals and oxidizing germ cells in the testicular tissue. Cisplatin (Cis) is a chemotherapeutic drug, a DNA-alkylating agent that exercises its anti-tumor function by prompting DNA crosslinks and DNA double-strand breaks; both destroy DNA transcription and replication, causing programmed cell death/apoptosis [4]. Moreover, Fallahzadeh et al. [5] stated that Cis triggers oxidative stress by generating reactive oxygen species (ROS) that stimulates cell destruction and necrosis through lipid peroxidation of tissues, protein denaturation, and DNA lesions.

Cisplatin (Cis) is one of the most widely used antitumor medications for the treatment of solid tumors, such as head and neck, testis, lung, ovarian, bladder cancer, and hematological malignancies [6], However, Cis’ use in clinical commitments is limited because of its side-effects, such as neurotoxicity, hepatotoxicity, and even testicular toxicity [5].

Cherry et al. [7] found that there was impaired fertility and decay in reproductive organ weights along with modifications in the growth and advancement of following generations in male rats treated with Cisp [7]. Both endocrine and exocrine compartments have an imbalance in spermatogenesis, gonadal dysfunction, androgenesis [8] and also prevent testosterone secretion by damage of Leydig cells. Cis decreases sperm motility and normal morphology. Chromosomal abnormalities in spermatozoa and azoospermia are related to the side-effects of Cis treatment. These side-effects are consigned to nitrosative and oxidative damage produced by Cis [9]. The physiological disturbance is due to increased lipid peroxidation and redox imbalance contributes to the apoptosis of the germ cells [10].

Recent studies have shown the antioxidant function of stem cells and their application to restore the damaging effects of ROS tissues [11]. Mesenchymal stem cells (MSCs) are multipotent stem cells originating from other tissues, such as the cord blood, adipose tissue, bone marrow, and amniotic membrane. MSCs are common in mature organisms and might be involved in tissue maintenance and repair, as well as in the regulation of immunological responses, function restoration, and homeostasis [12]. Squillaro et al. [13] stated that, in addition to chondrogenic, osteogenic, and adipogenic differentiation, MSCs are characterized by unique surface antigen expression; they are also able to self-renew themselves. Beside, stem cells will proliferate and create the daughter cell lines for the development of tissues [14]. The bone marrow is the source of MSCs from which to receive other tissues. Adult MSCs’ ability to ‘transdifferentiate’ could reform regenerative medicine [14]. Current studies have revealed the capacity of both embryonic and adult stem cells to distinguish into primordial germ cells and mature gametes. After the sixth day of birth, primordial germ cells (PGCs), the origin of male germ cells derived from the proximal epiblast, migrate to the seminiferous tubules’ basement membrane and become spermatogonial stem cells (SSCs) [15]. SSCs are responsible for sustaining the mechanism of spermatogenesis during its lifespan; the correct diagnosis of impaired spermatogenesis is a major stage in investigating male infertility. Nayernia et al. [15] proved that bone marrow stem cells are capable of distinguishing in vitro and in vivo between primordial germ cells and spermatogonia. Also, another study has confirmed that adult stem cells derived from the stroma bone marrow can differentiate into Leydig cells in rat testes [16].

Red beetroot (*Beta vulgaris*), a naturally occurring root vegetable, has numerous attentions as a health promoting functional food [17]. Beetroot regularly used up as part of the normal diet as a salad or fresh juice, and is used in the food industry as a food coloring agent recognized as E162 [18]. Recent studies have given convincing evidence that the ingestion of beetroot has beneficial physiological effects that could lead to enhanced clinical outcomes for many pathologies, such as hypertension, dementia, type II diabetes, and atherosclerosis. It is a rich source of phytochemical compounds that involves carotenoids, ascorbic acid, flavonoids, phenolic acids, and betalains [19]. Clifford et al. [20] well established the high antioxidant, anti-inflammatory, and anti-carcinogenic properties of betalains. Besides, beetroot has been reported to be mainly associated with human sex hormone enhancement as an aphrodisiac and also improves sexual weakness [21].

The goal of this work was to study the influence of mesenchymal stem cells and beetroot on improving testicular damage that cisplatin induced in rats.

## 2. Materials and Methods

The experiment was carried out in accordance with the guidelines of Al-Azhar University (Cairo, Egypt) for animal use. Rats were anesthetized by an i.p. injection of ketamine–xylazine (KX) combination at a dose of 50 mg/kg–6 mg/kg b.w. [22]. Xylazine was in the form of 2% Setone (Laboratorios Calier S. A., Les Franque-ses del Valles, Barcelona, Spain) and ketamine was in the form of Veta Ket^®^ injection 100 mg/mL ketamine hydrochloride (Lloyd, Shenandoah, IA, USA).

### 2.1. Materials

Cisplatin (Cis) (Sigma-Aldrich, St. Louis, MO, USA) was dissolved in phosphate-buffered saline (PBS) and the dose of Cis (7 mg/kg body weight) was selected on the base of previous works to induce testicular toxicity [5,23]. Fresh beetroot free from any physical defects was obtained from a local market in Cairo, Egypt.

### 2.2. Preparation of Extract

Fresh beetroots were washed with tap water to remove possible potential pathogenic microorganisms. Afterward, the beetroots were cut into small pieces of about 1 cm. Beetroot juice was prepared using a juicer and then filtered through cloth to obtain clear beetroot’s juice. This juice was concentrated in an oven under vacuum at a temperature of 50 °C until all water was extracted to give an extract sample. The dried extract was dissolved as a suspending agent in a mixture of carboxymethylcellulose and a few drops of Tween 80 to obtain 30 percent concentrated liquid extract (BRE) [24].

### 2.3. Stem Cells

Bone marrow mesenchymal stem cells (MSCs) were obtained from the Biochemistry and Molecular Biology Unit, Faculty of Medicine, Cairo University.

### 2.4. Experimental Design

For this study, 30 adults male Wistar albino rats were used (from the Helwan Serum and Antigen Laboratories). The rats weighing from 200–250 g were adapted to laboratory conditions for a one-week pre-experimentation period. They were kept in metabolic cages and provided fresh supplies of food daily and water ad-libitum. After one week acclimatization period, the rats were distributed into 5 groups of 6 animals each. The control group (NG) received saline; the Cisplatin group (CG) was injected with a single dose of 7 mg/kg i.p. to induce testicular toxicity; the Cis plus BM-MSCs treated group (CMG) injected with cisplatin (single dose 7 mg/kg, i.p.) and on the next day 2 × 10^6^ BM-MSCs in PBS by intravenous injection for one month [25]; Cis plus beetroot extract-treated group (CBG) treated with cisplatin (7 mg/kg, i.p.) and on the next day treated with 300 mg/kg b. wt/day of beetroot by oral gavage for one month [24]; Cis plus BM-MSCs plus beetroot extract combined group (CMBG) received cisplatin (7 mg/kg, i.p.) and 2 × 10^6^ BM-MSCs plus BRE (300 mg/kg b. wt/day) for one month. At the end of the experiment (over a month), the weights of the rats were in the range of 133–260 mg (the Cis group had a mean of 133 mg b.wt.).

### 2.5. Collection of Blood and Testicular Tissues

At the end of the experiment, the animals were anesthetized and blood samples obtained from the heart for testosterone assessment into heparinized tubes. Dissected and isolated testicular tissues from the scrotum were directly weighed. One testicle was soaked in 10% neutral formalin buffered after transverse cutting into two halves (used for histopathology and immuno-histochemistry). The other testicle was cut into two parts; one was homogenized and preserved at −80 °C for biochemical experiments, the other was placed in the Trizol reactive for gene expression analysis.

Estimation of the sperm count: Each epididymis was placed in a separate Eppendorf tube and gently squeezed into clean sterile watch glass and examined according to the technique adopted by [26] for the estimation of sperm count. Sperms were counted in average of two epididymis.

### 2.6. Evaluating the Levels of Serum Testosterone

The serum testosterone levels were calculated using ELISA testosterone kits (Abcam, Inc., Cambridge, UK) for rats, as instructed by [27]. The assay is based on competitive ELISA, where testosterone competes in the sample with the addition of testosterone-horseradish peroxidase (HRP) to bind the antibody. In brief, the anti-testosterone antibodies were pre-coated with a 96-well layer. Samples and testosterone-HRP conjugate were added to the wells, then the wells were washed and a substratum of 3,3′,5,5′-Ttetramethylbenzidine (TMB) was applied to create blue coloring. Stop Solution was added to avoid the production of colors and create a yellow color. The signal strength in the sample, which was calculated at 450 nm, was inversely proportional to the testosterone content.

### 2.7. Determination of Testicular Enzymes Levels

Succinate dehydrogenase activity was tested in rat testis using a colorimetric kit (ab228560) from Abcam. The activity of alkaline phosphatase was assayed calorimetrically in the testis of rats by the method prescribed by Piszczek et al., 2020 [28].

### 2.8. Assessment of Inflammation Markers

The degree of inflammation in serum samples was measured using the commercial Abcam’s Rat ELISA Kit (ab119548) to measure IL-6 levels.

### 2.9. Determination of Testicular Oxidative Stress Levels

Lipid peroxidation was evaluated in testicular tissue by colorimetric method using commercial kits according to the procedure of [29]. Testicular total antioxidant ability (TAC) and reduced glutathione (GSH) were calculated using commercial kits obtained from Bio Diagnostic Co., Dokki, Giza, Egypt.

### 2.10. Histological Examination of Testis

After 24 h, the 10% neutral buffered formalin preserved testicular tissues were washed under running tap water and preserved in 70% ethanol. The samples were dehydrated in ascending grades of ethanol, cleared in xylene, and embedded in Paraplast Plus^®^ (Leica Biosystems, Richmond, IL, USA), then cut at 5 µm thick sections for general histology and Immuno-histochemical techniques. For histological examination tissue sections were mounted on glass slides and stained with hematoxylin and eosin (H&E), then examined with a light microscope at 400× magnification power to assess any histological alterations. Photomicrographs were obtained with a light microscope 9 mega pixel.

### 2.11. Immuno-Histochemical Study

Immuno-histochemical studies were done on an average of 5 seminiferous tubules per slide, and 5 slides were used for each rat. Photomicrographs were obtained with a light microscope 9 mega pixel and analyzed with Image J program (image processing and analysis java 1.8.0_172). Degree of PCNA, or Caspase-3 staining were referred as mild staining (+), moderate staining (++), and strong staining (+++).

#### 2.11.1. Proliferating Cell Nuclear Antigen (PCNA)

The paraffin-embedded sections were assembled, deparaffinized, and washed with phosphate-buffered saline on charged sheets. Heating unmasked the antigen sites and inactivated the endogenous peroxidase by 3% H_2_O_2_. The sections were blocked for 1 h with 10% (*w*/*v*) natural goat serum and then incubated overnight at 4 °C with polyclonal rabbit anti-proliferating nuclear cell antigen (PCNA, Santa Cruz Biotechnology, Santa Cruz, CA, USA). All the samples were subsequently incubated at 37 °C for 30 min with biotinylated secondary antibodies (1:1000). The chromogenic 3, 3′-diaminobenzidine tetrachloride approach visualized the unique protein immunoreactivity Proliferating Cell Nuclear Antigen method.

#### 2.11.2. Caspase-3

The paraffin embedded sections were deparaffinized, rehydrated in a graded series of ethanol, and immersed in 3% H_2_O_2_ for 5 min twice. After 30 min, purified water was used to wash the sections, and a special glass pencil was used for drawing. After drying, 3% H_2_O_2_ was added for 10 min to inactivate the endogenous peroxidase, and the sections were then washed for 5 min with a phosphate-buffered solution (PBS, pH 7.2). Following this step, a protein block (compatible with both primary and secondary antibody) was used for almost 5 min to incubate and prevent non-specific ground staining. At the end of the incubation, the primary antibody was added (1.0% Caspase-3, Santa Cruz, CA, USA, control group PBS, Catalog number sc-9857). The sections were kept aside for 1 h and then subjected to a primer antibody at room temperature. PBS was used twice for washing the sections 5 min each time, and then the sections were incubated for 10–30 min with a secondary antibody at room temperature. The sections were kept in streptavidin–peroxidase, and PBS was again used in 10–30 min for the washing cycle. They were then subjected for 1–2 min to floor covering in counterstain Mayer’s Hematoxylin and washed with tap water. The lams were then held for 3 min in 80%, 96%, 100% ethanol, and xylol.

### 2.12. Gene Expression Analysis

Quantitative real-time PCR assay was applied to total RNA extracted from testicular tissue by a TRIzol reagent, according to the structure of the manufacturer to assess changes in the mRNA level of the inducible nitric oxide synthase (iNOS) gene in testicular tissues. A wavelength of 260 nm, concentration, and purity of extracted RNA was tested by Nanodrop. The integrity of total RNA was investigated by 1% agarose gel electrophoresis. To detect the change in caspase-3 (Casp3), steroidogenic acute regulatory protein (StAR), and inducible nitric oxide synthase (iNOS) genes expression, cDNA was synthesized using BioRad SYBER Green PCR Master Mix on Rotorgene RT-PCR system. The primer sequences were caspase-3 forward primer (F) 5′-CGATTATGCAGCAGCCTCAA-3′ and reverse primer (R) 5′-AGGAGATGCCACCTCTCCTT-3; StAR (F) 5′-GCAGCAGGCAACCTGGTG-3′ and (R) 5′-TGATTGTCTTCGGCAGCC-3; iNOS (F) 5-AAGAGTTCCCATCATTGCGT-3 and R 5-TCCTCAACCTGCTCCTCACT-3; GAPDH (P) 5′-TGT CGT GGA GTC TAC TGG TGT CTT-3′ and (R) 5′-CGT GGT TCA CAC CCA TCA CAA-3. The PCR reactions consisted of 95 °C for 10 min (one cycle), 94 °C for 15 s, and 60 °C for 1 min (40 cycles) [23]. Gene expression was quantified using GAPDH, a constitutively expressed housekeeping gene 2 (Delta Delta CT) method.

### 2.13. Statistical Analysis

Data were coded and entered using the Social Sciences Statistical Software (SPSS), version 25 (IBM Corp., Armonk, NY, USA). Data were summarized using mean and standard mean error (SE) for the quantitative variables. Comparisons were made using Variance Analysis (ANOVA) with several post-hoc comparisons for comparison between the two groups. *p*-values below 0.05 were found to be statistically significant.

## 3. Results

### 3.1. Changes in Sperm Count

**Figure 1:**
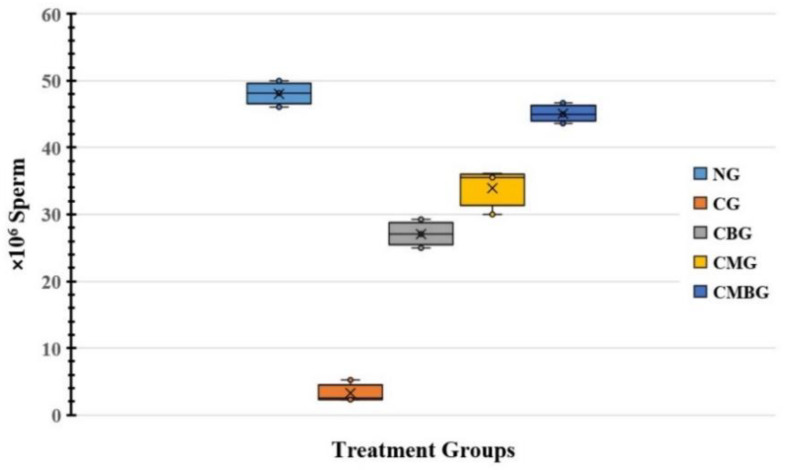
Box and whisker plot for sperm counts in the treated groups. The groups are: the control group (NG); the Cisplatin-treated group (CG); the Cisplatin+ beetroot extract-treated group (CBG); the Cisplatin+ mesenchymal stem cells-treated group (CMG); and the Cisplatin+ mesenchymal stem cells + beetroot extract-treated group (CMBG). The interrelations between all the presented groups are statistically significant (*p* ≤ 0.05). Group mean is presented by x symbols in the boxplots.

Treatment with Cis resulted in significant (*p* ≤ 0.05) decrease in sperm count (3.33 ± 1.45) relative to the control group (48.07 ± 1.79). The data also showed slight increase in sperm numbers corresponding to administration of BM-MSCs (2 × 10^6^ Cells) or beetroot extract (300 mg/kg body weight) with mean of 27.1 ± 1.92 and 33.87 ± 3.01, respectively. On the other hand, the Cis group treated with BM-MSCs combined with beetroot extract had a mean of 45.1 ± 1.39, which indicates significant improvement in sperm count, as compared to the control group (Figure 1 and Table 1).

### 3.2. Biochemical Results

#### 3.2.1. Serum Levels of Testosterone

**Figure 2:**
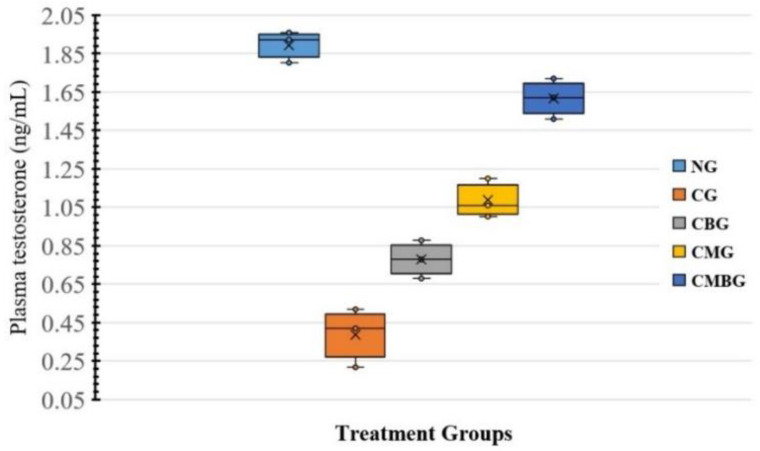
Box and whisker plot for serum testosterone concentration (ng/mL) in the treatment groups. The groups are: the control group (NG); the Cisplatin-treated group (CG); the Cisplatin+ beetroot extract-treated group (CBG); the Cisplatin+ mesenchymal stem cells treated group (CMG); and the Cisplatin+ mesenchymal stem cells + beetroot extract-treated group (CMBG). The interrelations between all the presented groups are statistically significant (*p* ≤ 0.05). Group mean is presented by x symbols in the boxplots.

Table 2 revealed that animals treated with cisplatin had significant reduction in the serum level of testosterone hormone (0.39 ± 0.1 ng/mL), compared to the control group (1.89 ± 0.07 ng/mL). However, supplementing the cisplatin group with either BM-MSCs (1.09 ± 0.09 ng/mL) or beetroot extract (0.78 ± 0.09 ng/mL) caused significant (*p* ≤ 0.05) improvement in testosterone level, compared to the cisplatin-treated group. The combination of both BM-MSCs and beetroot extract after Cisplatin enhanced the improvement (1.62 ± 0.09 ng/mL) (Figure 2).

#### 3.2.2. Testicular Enzymes Levels

**Figure 3:**
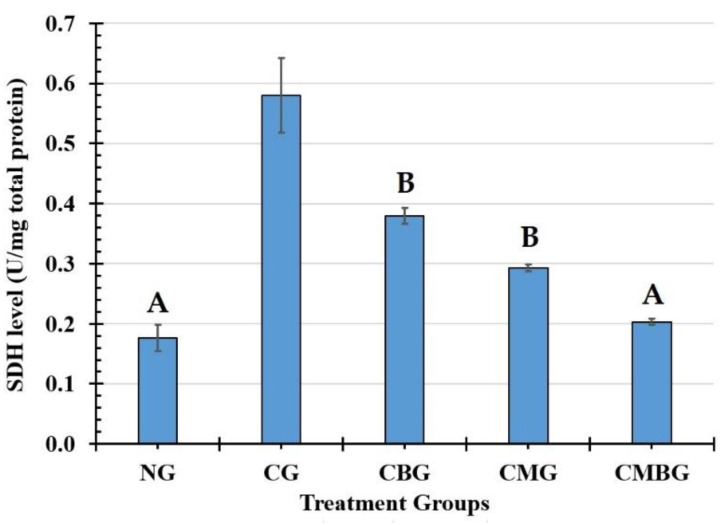
Histogram for succinate dehydrogenase (SDH) level (U/mg total proteins) in the treatment groups. The groups are: the control group (NG); the Cisplatin-treated group (CG); the Cisplatin+ beetroot extract treated group (CBG); the Cisplatin+ mesenchymal stem cells treated group (CMG); and the Cisplatin+ mesenchymal stem cells + beetroot extract treated group (CMBG). The interrelations between all the presented groups are statistically significant (*p* ≤ 0.05), except for the annotated groups (groups with the same letter have a statistically non-significant relationship).

**Figure 4:**
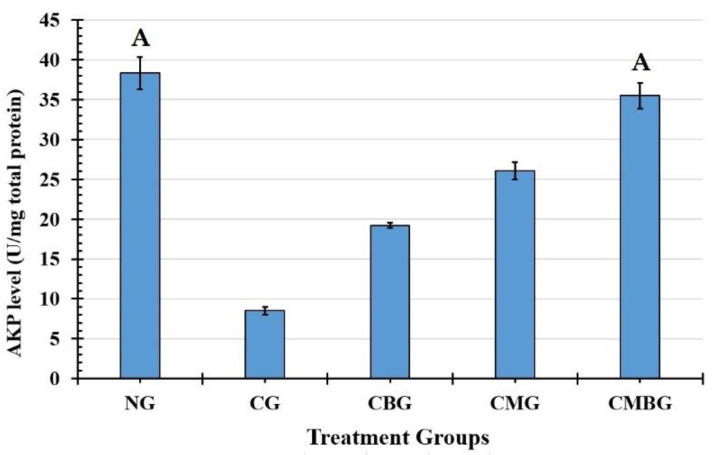
Histogram for alkaline phosphatase (AKP) level (U/mg total proteins) in the treatment groups. The interrelations between all the presented groups are statistically significant (*p* ≤ 0.05), except for the annotated groups (groups with the same letter have a statistically non-significant relationship). The groups are: the control group (NG); the Cisplatin-treated group (CG); the Cisplatin+ beetroot extract-treated group (CBG); the Cisplatin+ mesenchymal stem cells-treated group (CMG); and the Cisplatin+ mesenchymal stem cells + beetroot extract-treated group (CMBG).

The levels of testicular enzymes succinate dehydrogenase (SDH) and alkaline phosphatase (AKP) are illustrated in Figure 3. Compared with the control group (0.18 ± 0.02 U/mg total protein), the level of SDH increased significantly (*p* ≤ 0.05) after induction with Cis (0.58 ± 0.06 U/mg total protein). After treatment with either BM-MSCs or beetroot separately or in concordance, SDH activity was reduced without noticeable difference between the three groups (Table 3). AKP displayed a significant (*p* ≤ 0.05) decrease in the Cis-treated group (8.50 ± 0.52 U/mg total protein) as compared to the control group (38.33 ± 2.02 U/mg total protein). However, AKP increased significantly (*p* ≤ 0.05) in the groups treated with Cis, followed by BM-MSCs or beetroot (Figure 4). Co-treatment with BM-MSCs and beetroot produced a significant (*p* ≤ 0.05) induction of AKP activity (35.50 ± 1.60 U/mg total protein) than the single treated groups (Table 3).

#### 3.2.3. Cytokine Interleukin-6 (IL-6) Levels

**Figure 5:**
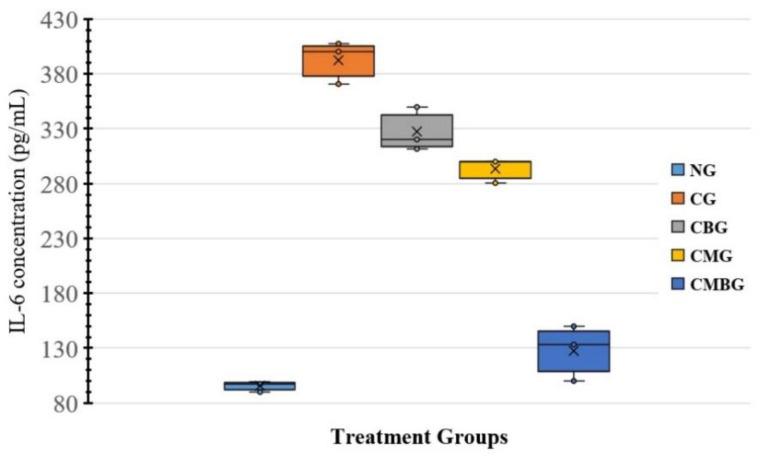
Representation for Interleukin-6 (IL-6) concentration (ng/mL) in the treatment groups. The groups are: the control group (NG); the Cisplatin treated-group (CG); the Cisplatin+ beetroot extract-treated group (CBG); the Cisplatin+ mesenchymal stem cells-treated group (CMG); and the Cisplatin+ mesenchymal stem cells + beetroot extract-treated group (CMBG). The interrelations between all the presented groups are statistically significant (*p* ≤ 0.05).

The levels of pro-inflammatory (IL-6) cytokines (Table 4) revealed significant increase (*p* ≤ 0.05) in the Cis-treated group (392.7 ± 17.19 pg/mL), as compared to the control group (95.43 ± 4.28 pg/mL). However, IL-6 was significantly decreased (*p* ≤ 0.05) in the Cis group treated with BM-MSCs only (293.33 ± 10.1 pg/mL) or beetroot only (327.4 ± 17.92 pg/mL). Co-treatment with BM-MSCs and beetroot created markedly significant (*p* ≤ 0.05) decrease (127.8 ± 22.78 pg/mL) over the Cis group (Figure 5).

#### 3.2.4. Oxidative Stress Levels

**Figure 6:**
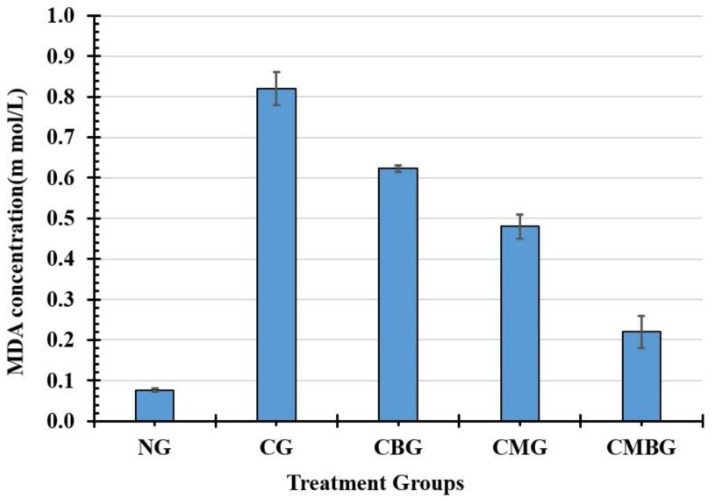
Histogram for testicular malondialdehyde (MDA) concentration (m mol/L) in the treated groups. The interrelations between all the presented groups are statistically significant (*p* ≤ 0.05). The groups are: the control group (NG); the Cisplatin-treated group (CG); the Cisplatin+ beetroot extract-treated group (CBG); the Cisplatin+ mesenchymal stem cells-treated group (CMG); and the Cisplatin+ mesenchymal stem cells + beetroot extract-treated group (CMBG).

Physiological values of MDA, GSH, and TAC activity in testicular injury caused by cisplatin are identified in Table 5. In the cisplatin group, MDA levels (0.82 ± 0.04 m mol/L) showed statistically significant (*p* ≤ 0.05) increase, compared to the control group (0.08 ± 0.01 m mol/L), but treatment with BM-MSCs or beetroot significantly (*p* ≤ 0.05) reduced the concentrations of MDA levels in comparison to the cisplatin and control values of this parameter (Figure 6).

TAC levels’ glutathione (GSH) activity was compared to the cisplatin group: the activity of the enzyme increased slightly in the BM-MSCs or beetroot group towards the normal level (control group). However, the extreme correction effect was reported in cisplatin rats treated with BM-MSCs in combination with beetroot.

**Figure 7:**
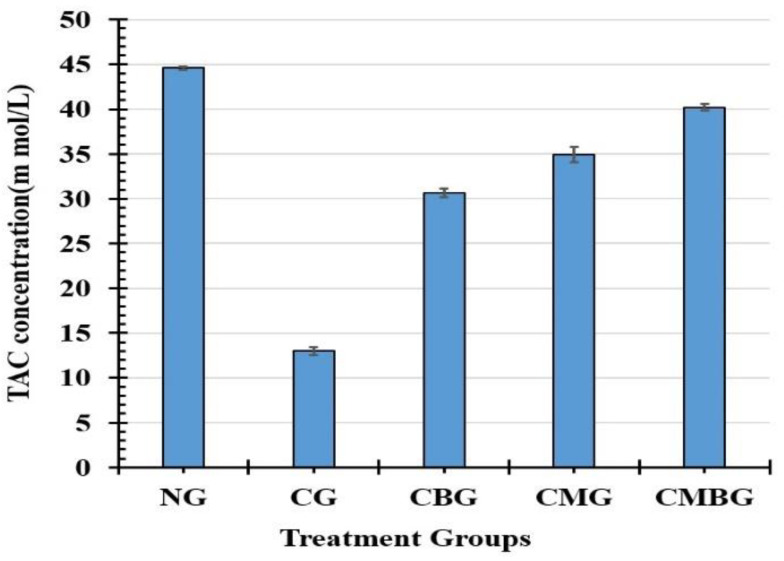
Histogram for testicular total antioxidant capacity (TAC) concentration (m mol/L) in the treated groups. The interrelations between all the presented groups are statistically significant (*p* ≤ 0.05). The groups are: the control group (NG); the Cisplatin-treated group (CG); the Cisplatin+ beetroot extract-treated group (CBG); the Cisplatin+ mesenchymal stem cells-treated group (CMG); and the Cisplatin+ mesenchymal stem cells + beetroot extract-treated group (CMBG).

TAC level (Figure 7) was maximum in the control group (44.59 ± 0.22 m mol/L), followed by the CMBG (40.21 ± 0.38 m mol/L), the CMG (34.9 ± 0.85 m mol/L), the CBG (30.64 ± 0.50 m mol/L), and the minimum level in the Cis group (12.99 ± 0.47 m mol/L). All relationships between mean of groups recorded statistically significant (*p* ≤ 0.05) differences with least significant difference of 0.747.

**Figure 8:**
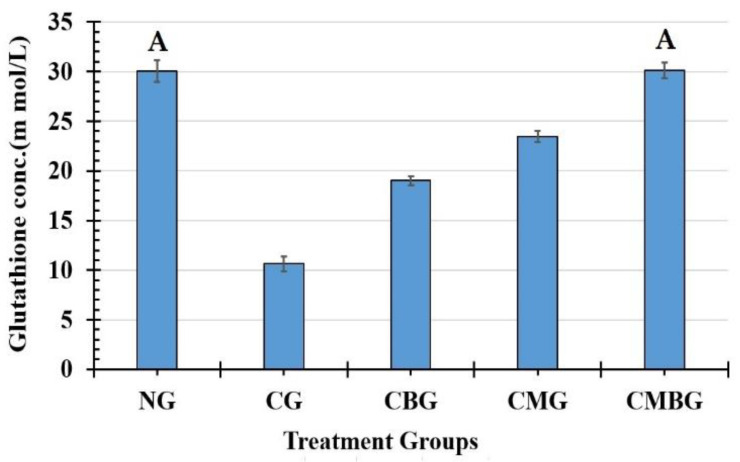
Histogram for glutathione concentration (m mol/L) in the treated groups. The interrelations between all the presented groups are statistically significant (*p* ≤ 0.05), except for the annotated groups (groups with same letter have insignificant relation). The groups are: the control group (NG); the Cisplatin-treated group (CG); the Cisplatin+ beetroot extract-treated group (CBG); the Cisplatin+ mesenchymal stem cells-treated group (CMG); and the Cisplatin+ mesenchymal stem cells + beetroot extract-treated group (CMBG).

The GSH activity level had a pattern similar to that of TAC with maximum level in the CMBG (30.13 ± 0.80 m mol/L) followed by the normal group (30.03 ± 1.07 m mol/L), CMG (23.47 ± 0.55 m mol/L), CBG (19.03 ± 0.45 m mol/L), and Cis group (10.63 ± 0.75 m mol/L). All groups have statistically significant difference between means, except for the relationship between the control group and the CMBG (Figure 8). The least significant difference between means in all groups was 1.071.

### 3.3. Histopathological Analysis

#### 3.3.1. Haematoxylin and EOSIN (H&E)

**Figure 9:**
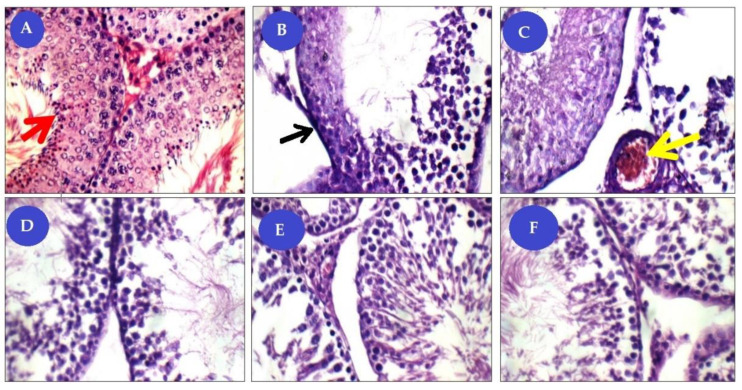
Panel of histological (H&E ×400) pictures showing: (**A**) control group (NG) seminiferous tubules (ST) with average BM (basement membrane), spermatogonia, primary spermatocyte, and many spermatozoa (red arrows); (**B**,**C**) Cisplatin-treated group (CG) with tubules have detached and thick BM (black arrow), marked reduction of germinal lining with few sperms, and interstitial blood vessels congested (yellow arrow). (**D**) Cisplatin+ mesenchymal stem cells treated group (CMG) tubules with mildly thick BM, mild reduction of germinal lining with moderate number of sperms. (**E**) Cisplatin+ mesenchymal stem cells+ beetroot extract treated group (CBG) tubules with average BM, average germinal lining up to full spermatogenesis, and average interstitium with average Leydig cells. (**F**) Cisplatin+ mesenchymal stem cells+ beetroot extract treated group (CMBG) tubules with mildly thick BM, average germinal lining up to full spermatogenesis, and average interstitium with average Leydig cells.

Light microscopic analysis of the test parts in the standard control group revealed a dense connective tissue capsule containing the testis. The seminiferous tubules and interstitial tissue form the structural elements from the testicle. The seminiferous tubules lined with a germinal epithelium resting on a basement membrane and included the normal arrangement of spermatogenic layers, spermatogonia, primary spermatocyte, and many spermatozoa (Figure 9A). However, the Cis-treated rats showed signs of testicular dis-functions involving failure production of sperms and maturation arrest during the process of spermatogenesis and marked histological lesions (Figure 9B,C). Most observed histopathological examinations have a marked decrease in the thickness of the germ layers, an apparent decrease in the number of spermatogenic cells. On the other hand, after treatment, testis of CBG and CMG showed a mild reduction of germinal lining with a moderate number of sperms figures (Figure 9D,E). Meanwhile, in the combined treatment (CMBG), testis showed average tunica albuginea, mildly congested sub-capsular blood vessels, and average-sized tubules with mild reduction of the germinal lining in some of the tubules with complete spermatogenesis, and interstitium showed increased numbers of Leydig cells (Figure 9F).

#### 3.3.2. Immuno-Histochemical Studies

##### Immuno-Staining for the Proliferating Cell Nuclear Antigen (PCNA)

**Figure 10:**
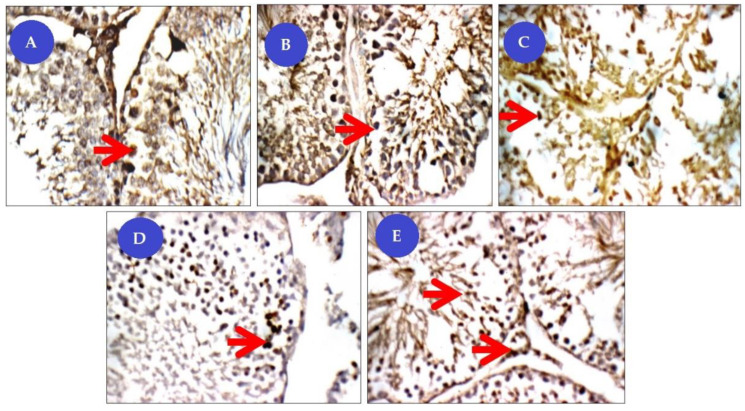
Panel of proliferating cell nuclear antigen immuno-histochemical (IHC) pictures (×400). Degree of PCNA staining were referred to as mild staining (+), moderate staining (++), and strong staining (+++). (**A**) control group (NG) testicular tissue with mild nuclear reactivity (++) for PCNA in basal tubular lining, spermatogonia (red arrow) and primary spermatocytes; (**B**) Cisplatin-treated group (CG) has marked low nuclear reactivity (+) for PCNA in tubular lining from spermatogonia (red arrow) up to spermatids, and in interstitial cells; (**C**) Cisplatin+ beetroot extract treated group (CBG) with marked nuclear reactivity (++) for PCNA in tubular lining from spermatogonia (red arrow) up to spermatids, and in interstitial cells; (**D**) Cisplatin+ mesenchymal stem cells treated group (CMG) has moderate nuclear reactivity (++) for PCNA in tubular lining from spermatogonia (red arrows) up to spermatids; (**E**) Cisplatin+ mesenchymal stem cells+ beetroot extract treated group (CMBG) has marked more nuclear reactivity (+++) for PCNA in tubular lining from spermatogonia (red arrow) up to spermatids, and in interstitial cells.

In all possible cases, nuclear staining was expressed as brown color fine dots (Figure 10). In the control group, the testes showed mild expression of PCNA positive immune reactivity (++), where the nucleus appeared brownish in color. However, in the Cis group, the PCNA immune reactive cells of testis showed a low number of PCNA positive nucleus (+). Groups of Cis rats treated with BM-MSCs or beetroot extract showed more expression and widespread PCNA positive nucleus (++) than Cis non-treated rats. In particular, the expressions of immune reactivity of the PCNA significantly improved (+++) in the nuclei of basal tubular lining, spermatogonia, and primary spermatocytes rates concurrently treated with BM-MSCs and beetroot extract.

##### Immuno-Staining for Caspase-3

**Figure 11:**
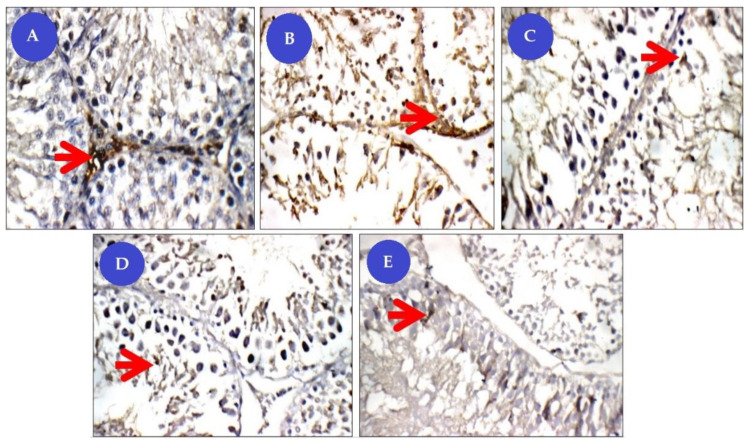
Panel of Caspase-3 immune-histochemical (IHC) pictures (×400). Degree of caspase staining were referred to as mild staining (+), moderate staining (++), and strong staining (+++). (**A**) control group (NG) testicular tissue showing mild cytoplasmic reactivity (+) for Caspase-3 in basal tubular lining, spermatogonia (red arrow), and primary spermatocytes; (**B**) Cisplatin-treated group (CG) with more cytoplasmic reactivity (+++) for Caspase-3 in tubular lining from spermatogonia (red arrow) up to spermatids; (**C**) Cisplatin+ beetroot extract treated group (CBG) with mild cytoplasmic reactivity (++) for Caspase-3 in tubular lining, from spermatogonia (red arrows) up to spermatids, and in interstitial cells; (**D**) Cisplatin+ mesenchymal stem cells treated group (CMG) showing mild cytoplasmic reactivity (++) for Caspase-3 in tubular lining from spermatogonia (red arrow) up to spermatids, and in interstitial cells; (**E**) Cisplatin+ mesenchymal stem cells+ beetroot extract treated group (CMBG) figuring low nuclear reactivity (+) for Caspase-3 in tubular lining from spermatogonia (red arrow) up to spermatids, and in interstitial cells.

Immuno-staining for Caspase-3 was expressed as brown color in the cytoplasm. The spermatogonia up to spermatids, and in interstitial cells showed low expression of Caspase-3 positive immune reactivity (+) where cytoplasm appeared brownish in the control group. The Cis group showed high immune reactive cells (+++) of the testes, while the group of Cis rats treated with BM-MSCs or beetroot extract showed a moderate increase (++). On the other hand, the Cis group concurrently treated with BM-MSCs and beetroot extract showed lower expression of positive immune reactivity (+) of caspase-3 in relation to Cis-untreated rats (Figure 11).

### 3.4. Gene Expression

The current experiment used real-time PCR to detect the mRNA expression levels of three genes as different molecular biomarkers utilizing GAPDH as a reference housekeeping gene.

#### 3.4.1. iNOS Gene mRNA Expression Analysis

**Figure 12:**
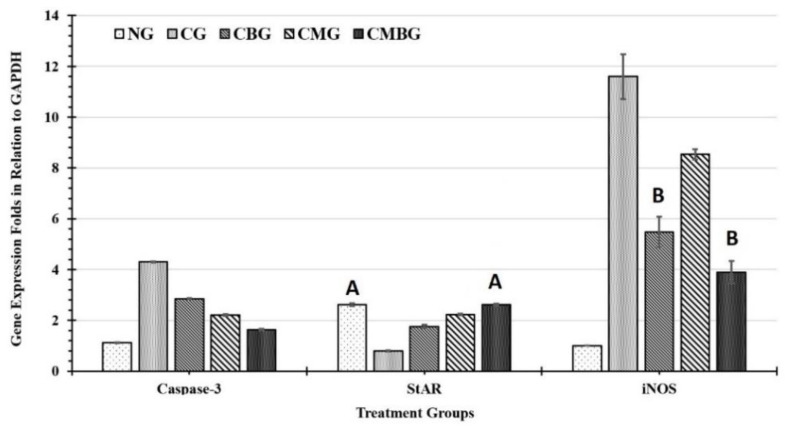
Histogram for gene expression in response to the treatments. The genes were caspase-3; steroidogenic acute regulatory protein (StAR); and inducible nitric oxide synthase (iNOS). The groups are: the control group (NG); the Cisplatin treated group (CG); the Cisplatin+ beetroot extract treated group (CBG); the Cisplatin+ mesenchymal stem cells treated group (CMG); and the Cisplatin+ mesenchymal stem cells + beetroot extract treated group (CMBG). The interrelations between all the presented groups are statistically significant (*p* ≤ 0.05), except for the annotated groups (groups with the same letter have insignificant relationship).

The inflammatory impact of cisplatin on testicular tissues was detected in the statistically significant (*p* ≤ 0.05) increase of iNOS gene expression (11.6 ± 0.89), compared with the control group (1 ± 0.00). The protective effect of BM-MSCs, as well as beetroot extract separately was demonstrated by the significant reduction of the iNOS gene expression level (8.54 ± 0.2 and 5.48 ± 0.6, respectively) compared to the Cis group. Whereas, the combination of BM-MSCs+ beetroot extract revealed the highest protective role (3.9 ± 0.44) in the modulation of cisplatin testicular injury in comparison with all groups (Figure 12). All current experiment groups showed statistically significant (*p* ≤ 0.05) intergroup difference except for group CBG with CMBG. The calculated least significant difference between the groups was 0.742 (Table 6).

#### 3.4.2. Caspase Gene Expression Analysis

The apoptotic biomarker caspase-3 has maximum induction at the positive control group after treatment with Cis (4.31 ± 0.02), as compared to the current groups. However, treatment with stem cells and beet root produced maximum inhibition of gene expression at the mRNA level (1.64 ± 0.03) between the groups (Figure 12). Treatment with stem cells alone or beet root extract alone after caspase induction with Cis (CMG) resulted in statistically significant (*p* ≤ 0.05) decrease in caspase mRNA expression (2.21 ± 0.04 and 2.85 ± 0.02, respectively) as compared to the positive control (CG) and the negative control (untreated group; NG = 1.13 ± 0.01). All current experiment groups have statistically significant (*p* ≤ 0.05) differences in between with least value (LSD) of 0.035.

#### 3.4.3. StAR Gene Expression Analysis

The fertility molecular biomarker StAR gene has current mRNA expression of 2.62 ± 0.05 in the control untreated group (NG). It was decreased to the minimum level in the Cis-treated group (0.80 ± 0.02), as compared to all the other current groups (Figure 12). The StAR mRNA expression level was normalized (2.62 ± 0.04) when treated with stem cells and beetroot extract after Cis exposure (CMBG) as compared to the positive (CG) and negative (NG) control groups. However, stem cell alone after Cis exposure CMG, 2.23 ± 0.03) was more effective than beetroot extract treatment after cis (CBG, 1.76 ± 0.06) in elevating the mRNA level of StAR. All groups showed statistically significant differences (*p* ≤ 0.05) in between, except for the negative control group (NG) and the CMBG. The value of the least significant difference (LSD) was 0.035 (Table 6).

## 4. Discussion

Dysfunction or testis damage are the serious side-effects of chemotherapy drugs such as cisplatin. In the present study, we estimate the sperm count, several biochemical, hormonal, histological, and gene expression parameters related to testicular toxicity and oxidative stress in the testis tissue to evaluate the protective effect of BM-MSCs and beetroot against Cis-induced reproductive toxicity in rats. Cis treatments cause cytotoxicity as ROS formation, DNA damage leading to germ epithelial damage, sperm dysfunction, and germ cells apoptosis. It also leads to inflammation [7].

In the present investigation, Cis administration contributed to a drop in sperm count as a result of cisplatin toxic side-effects, resulting in a reduced testicular activity. The free radical development and decreasing cisplatin-induced antioxidant enzymes cause rapid loss of intracellular adenosine triphosphate (ATP), leading to loss of sperm motility and reduced sperm viability [30]. In addition, reducing sperm motility and increasing abnormal sperm rate in rats treated with cisplatin could be caused by lipid peroxidation of unsaturated fatty acids in the sperm plasma membrane, resulting in loss of fluidity and function [31].

Following the treatment of the Cis group with BM-MSCs and beetroot extract, there was a statistically significant improvement in sperm count. These findings were in accordance with Dillasamola et al. [32], who found that beetroot extract restores natural sperm count and motility after xenobiotic toxicity. Beetroot’s mechanism of action can serve as a useful approach to reinforce endogenous antioxidant defenses and protect cellular constituents from xenobiotics [33].

BM-MSCs are confirmed to have been used in experimental rodent infertility models treated with busulfan [16]. In the current work, the MSC group was correlated with significantly higher sperm compared to the rat group treated with Cis. Hence, it could be concluded that MSCs might have a potential role in treating male infertility and testosterone deficiency. It could also be suggested that the beneficial effects of MSCs may be due to differentiation in male germ cells, as stated by Nayernia et al. [15]. Yazawa et al. [16] established that MSCs are able to differentiate into steroidogenic cells, such as Leydig cells, in vivo as well as in vitro.

Testosterone plays an important role in controlling spermatogenesis. Concurrent tests after cisplatin administration indicated a substantial decrease in serum testosterone levels. Recent research showed that cisplatin harms the testis with decreased testosterone levels [5]. This remarkable reduction in hormonal level may be explained by the serious damage caused by cisplatin on Leydig and Sertoli cells as one of the possible mechanisms resulting from the increased generation of free radicals [34]. Moreover, several researchers have reported that interference with the expression of the Luteinizing Hormone (LH) receptor and cholesterol mobilization inhibition to mitochondrial cytochrome P450 interferes with the initial steps in testosterone production. Such results are consistent with those suggested by [34].

The administration of BM-MSCs and beetroot extract to Cis-treated animals led to significant increase in serum testosterone concentration, which corresponded to Mohammadi et al. [35], who informed that improvement in testosterone level achieved by beetroot extract was due to its antioxidant activity. Regarding BM-MSCs, this was used as a therapeutic tool and elevated levels of testosterone, leading to the transformation of MSCs into steroid cells as Leydig cells containing testosterone [14].

In the testis, AKP is associated with sperm cell division and glucose transfer to spermatogenic cells and SDH, biomarkers of the testicular enzyme that relate to sperm metabolism. SDH is widely distributed and found in germ cells and seminiferous tubules, which relate to sperm cell maturation, testis, and spermatozoa, and the spermatozoa energy metabolism [36]. The present data shows the levels of testicular AKP and SDH significantly decreased or increased in the Cis groups, which indicates that Cis may influence sperm cell energy metabolism. In the current research, co-administration with BM-MSCs and beetroot extract to the Cis group induced improvement in the function of those enzymes.

Cytokines perform important roles in normal cell physiology. These are linked to or healed by inflammation, immune response, and tissue damage. Cisplatin stimulated inflammatory cells and consequently magnified the inflammatory response by releasing various cytokines such as TNF and IL-6, resulting in damage to the testes. In this study, the treatment of rats with BM-MSCs combined with beetroot significantly reduced serum levels of IL-6. Therefore, it may be recommended that BM-MSCs with beetroot may relieve cisplatin-caused testis injury by suppressing the inflammatory response. In addition, these results were in agreement with Winkler et al. [37] who reported beetroot extract to be capable of counteracting proinflammatory cascades in the periphery mononuclear blood cells [37]. Due to the strong role of inflammation in the development and progression of many clinical conditions, including coronary heart disease and cancer, the beneficial effect of beetroot extract may be associated with this anti-inflammatory capability [38]. Therefore, BM-MSCs with beetroot may be suggested to mitigate cisplatin-caused injury to the testis by suppressing the inflammatory response. Albasher et al. [39] mentioned that beetroot treatment reduced TNF-α and IL-6 in renal tissue exposed to gentamicin by the inactivation of NF-κB.

Previous studies have shown that Cis toxicity induces oxidative stress, leading to the generation of free radicals with an increase in ROS and reduced antioxidant molecules of the enzymatic and non-enzymatic testicular protection. In the current study, Cis showed that rat’s antioxidant profile changed as testicular MDA increased and TAC decreased, with GSH demonstrating that enzymatic and non-enzymatic antioxidant molecules were insufficient to scavenge free radicals generated from cisplatin [40]. The increase in the amount of MDA may be attributed to the excess output of ROS attacking the cell membrane due to a deficiency of antioxidant enzymes [41]. The decrease in these enzymes’ activity could be explained either by their consumption during the transformation of free radicals into less hazardous or harmless metabolites, or by the direct inhibitory effect of cisplatin on the enzyme function [42]. The testis is one of the main target organs for oxidative stress and peroxidative destruction due to the high polyunsaturated fatty acids and low antioxidant concentration efficiency, which results in reduced sperm vitality, motility, and consequently infertility [43].

Elshiekh et al. [44] reported results on beetroot improvements to cisplatin damage in rats; these were in line with the current work. They recorded significant improvements in sperm count, sperm morphology, histopathology, serum testosterone, MDA level, glutathione (GSH), and catalase activities [44].

The administration of BM-MSCs and beetroot extract to the Cis group in the current study induced significant improvements in the lipid peroxidation marker and antioxidant enzymes (TAC and GSH). These results agreed with Lorizola et al. [45], who indicated that the administration of beetroot juice (BRJ) shows a substantial decline in the toxic carbon tetrachloride group for serum MDA levels. Interestingly, apigenin-derived flavonoid glycosides may have acted as natural exogenous antioxidants in beet stalks and leaves, reducing or decreasing oxidative stress in mice. Beetroot is moreover an extremely rich source of antioxidants. Several studies have shown that betalain pigments, in particular, protect cellular components against oxidative damages [46]. The disturbance in prior oxidative stress levels was reversed after the injection of MSCs. Such results agree with other studies that demonstrated the antioxidant activities of MSCs [47].

Great improvement was noticed in combined BM-MSCs and beetroot extract to Cis toxic group. The histopathological lesions observed in the present results are in corroboration with the observed biochemical and cytogenetic changes. The number of spermatogenic cells is decreased and the seminiferous tubule with vacuoles underwent degenerative changes in the Cis group. It has been documented that mesenchymal stem cells (MSCs) were able to identify and migrate to injury sites when transplanted systemically, indicating they had migratory ability. Homing to the damaged site and subsequently differentiating into tissue-specific cells, MSCs act as an essential tissue member and thus contribute to tissue repair [48].

In addition, the ameliorative effect of MSCs may be by preventing testicular apoptosis, reducing intra-testicular oxidative stress, and promoting the development of testosterone which stimulates spermatogenesis. In previous research, this suggested the impact of MSC on ischemic injury in models of the kidney, heart, and lung [49].

Our immunohistochemical studies revealed that the Cis group showed significantly increased anti-caspase expression−3 in the spermatogenic cell cytoplasm, which plays a vital role in seminiferous tubules in apoptosis regulation [50]. Increased caspase activity in the testis was also associated with various pathologies such as impaired spermatogenesis, fragmentation of sperm DNA, reduced sperm motility varicocele, immune infertility, and testicular torsion. It serves as a junction point for different signaling pathways in the execution of cell apoptosis. Its activation leads to DNA degradation, chromatin condensation, and membrane protein destruction. Cis administration was shown to result in upward control of caspase-3 and germ cells and Leydig cell apoptosis by triggering extrinsic, intrinsic, and other metazoan apoptotic pathways [51]. These results were due to the oxidative stress condition caused by the administration of cisplatin and its concomitant impact on the apoptosis of germ cells. Therefore, BM-MSCs with beetroot can be suggested to attenuate the level of caspase-3 immunoreactivity in the testes. The BM-MSC defense mechanism of action is believed to be mainly due to the ability of MSCs to defend testis against oxidative stress through their antioxidant ROS-scavenging properties [52]. Besides, MSCs can modify the immune and inflammatory state induced by Cis administration [53]. MSC ultimately exercised anti-apoptotic trophic, and tissue regeneration properties. In this sense, MSCs will stimulate the remaining sperm cells to proliferate and complete their division, releasing some growth factors and cytokines [54]. This was also confirmed by our immunohistochemical tests, which demonstrated weak caspase-3 expression in the Cis treated group with a combination of BM-MSCs and beetroot extract.

PCNA is an intranuclear polypeptide and a cofactor of DNA polymerase delta, essential for repair, excision, and replication [55]. As spermatogenesis is a complex cell cycle of fast proliferating cells ending with the formation of sperms, we used PCNA in this study as a tool to evaluate spermatogenesis. We observed that PCNA positive cells were strongly expressed in spermatogonia and early-stage spermatocytes of normal rat, whereas, in Cis-untreated rats, the number of PCNA positive testicular germ cells was substantially reduced, which is an indicator of disturbance in proliferation and spermatogenesis. Earlier studies have suggested that increased PCNA expression in testicular tissue indicates high proliferative activity and stimulation of the sperm [56]. This study showed that the adverse effect of lowered PCNA expression in the Cis group is improved by treatment with a combination of BM-MSCs and beetroot extract. Thus, the upregulation of PCNA led to the promotion of cell cycle progress and the decline of apoptosis.

Inducible Nitric Oxide Synthase (iNOS) is one of three known isoforms responsible for the synthesis of nitric oxide (NO) in mammalian nitric oxide synthase (NOS). The main iNOS control mechanism seems to be at the genetic induction level, hence its common name as an inducible enzyme [57]. It is responsible for the production of high levels of nitric oxide (NO) under oxidative stress, which results in auto-cytotoxicity [58]. Studies have demonstrated that NO’s oxidation products induce lipid peroxidation [59]. Transcription factors such as NFkB stimulated under oxidative stress can also prompt the expression iNOS. Blocking NF-kB and therefore iNOS with antioxidants has already been shown to be effective in alleviating testicular injury caused by Cis [60]. Numerous studies have shown that nitric oxide reacts with superoxide anion, forming radical peroxynitrite, which therefore oxidizes cellular structures and induces lipid peroxidation [58]. It is extremely toxic, causing oxidizing damage to cell components, including lipids, nucleic +acids, and proteins.

The research results suggest that cisplatin significantly increased the level of iNOS gene mRNA. Additionally, MSC and beetroot extract treatment of cisplatin-induced damaged testicular tissues causes substantial diminution of the iNOS gene regulation relative to cisplatin-treated rats. This result suggests that the protective effect of BM-MSCs and beetroot extract may be closely linked to its cisplatin-induced inhibition of nitric oxide production. Some earlier studies have stated the possible antioxidant properties of both beetroot and MSC against several forms of free radicals [61].

One of the most important molecular biomarkers of fertility is the steroidogenic acute regulatory (StAR) protein. It is responsible for regulation of steroid hormone biosynthesis in steroidogenic cells of testis. The StAR protein is important to transport cholesterol from the outer membrane to the inner side of the mitochondria [62]. StAR protein expression is regulated by LH-mediated activation of cAMP-dependent pathways, leading to transcriptional activation in Leydig cells [63]. StAR expression is correlated to stress on testis, which affect its efficiency [64,65,66]. The current results recorded an improvement in testis efficiency in the groups treated with stem cells and beetroot extract after Cis treatment considering the expression levels of the StAR gene.

Caspase-3 is a member of intrinsic protein factors for programmed cell death (apoptosis). Inactivation of caspase-3 dramatically reduces apoptosis in diverse settings, including activation-induced cell death (AICD). It is an essential component in apoptotic events that are remarkably system- and stimulus-dependent [67]. The current results indicated overexpression in caspase-3 after treatment with Cis, which activates apoptosis. However, the current cell therapy combined with beetroot extract was effective in improvement of Cis side-effects. This can be explained by previous studies, which stated that dying cells provide instructive cues that can influence surrounding cells to proliferate and showed that even dying stem cells facilitate communication with adjacent stem cells by caspase-dependent production of Wnt8a-containing apoptotic bodies to drive cellular turnover [68].

## 5. Conclusions

There was significant improvement in testis function assays, fertility, and testicular histopathology on combination of stem cell therapy with natural products utilizing BM-MSCs plus beetroot extract after side-effects of Cis treatment in rats.

This study confirmed that cisplatin induced testicular damage. BM-MSCs and beetroot extract have antioxidant effectiveness, anti-inflammatory efficacy, and prevent oxidative stress. Therefore, the current study planned to investigate the potential defensive effects of BM-MSCs and beetroot extract on cisplatin-induced testicular damage in rats.

The current study proposes the use of BM-MSCs supported with beetroot to overcome the expected infertility side-effect of Cis chemotherapy.

## Figures and Tables

**Table 1:** Changes in epididymis sperm count in the control and experimental groups.

Group	Sperm Count × 10^6^
NG	48.07 ± 0.73
CG	3.33 ± 0.59
CBG	27.1 ± 0.79
CMG	33.87 ± 1.23
CMBG	45.1 ± 0.57
LSD	1.154

Data are expressed as mean for six-sampled group ±SE. All groups have statistically significant (*p* ≤ 0.05) difference between means. The groups are: the normal control group (NG); the Cis group (CG); the group treated with Cis+ beetroot extract (CBG); the group treated with Cis+ mesenchymal stem cells (CMG); and the group treated with Cis+ mesenchymal stem cells + beetroot extract (CMBG). The least significant difference between means (LSD = 1.154).

**Table 2:** Changes in serum levels of testosterone in the control and experimental groups.

Group	Serum Testosterone (ng/mL)
NG	1.89 ± 0.03
CG	0.39 ± 0.06
CBG	0.78 ± 0.04
CMG	1.09 ± 0.04
CMBG	1.62 ± 0.04
LSD	0.057

Data are calculated as the mean for six-sampled group ± SE. The groups are: the normal control group (NG); the Cis group (CG); the group treated with Cis+ beetroot extract (CBG); the group treated with Cis+ mesenchymal stem cells (CMG); and the group treated with Cis+ mesenchymal stem cells + beetroot extract (CMBG). All groups have statistically significant (*p* ≤ 0.05) difference between means. The least significant difference between means (LSD = 0.57).

**Table 3:** Changes in testicular enzyme levels in the control and experimental groups.

Group	SDH (U/mg Protein)	AKP (U/mg Protein)
NG	0.18 ± 0.02 A	38.33 ± 2.02 A
CG	0.58 ± 0.06	8.50 ± 0.52
CBG	0.38 ± 0.01 B	19.23 ± 0.31
CMG	0.29 ± 0.01 B	26.07 ± 1.10
CMBG	0.20 ± 0.01 A	35.50 ± 1.60 A
LSD	0.043	1.81

Data are calculated as mean for the six-sampled group ± SE. All groups have statistically significant (*p* ≤ 0.05) difference between means, except for groups with the same letter in the same column. The enzymes are succinate dehydrogenase (SDH) and alkaline phosphatase (AKP). The groups are: the normal control group (NG); the Cis group (CG); group treated with Cis+ beetroot extract (CBG); the group treated with Cis+ mesenchymal stem cells (CMG); and the group treated with Cis+ mesenchymal stem cells + beetroot extract (CMBG). The least significant difference (LSD) values were 0.043, and 1.81.

**Table 4:** Changes in Interleukin-6 levels in sera of the control and experimental groups.

Group	IL-6 (pg/mL)
NG	95.43 ± 1.75
CG	392.7 ± 7.02
CBG	327.4 ± 7.32
CMG	293.33 ± 4.12
CMBG	127.8 ± 9.3
LSD (Least Significant Difference)	9.15

Data are calculated as mean for six-sampled group ± SE. All groups have statistically significant (*p* ≤ 0.05) difference between means. The groups are: the normal control group (NG); the Cis group (CG); the group treated with Cis+ beetroot extract (CBG); the group treated with Cis+ mesenchymal stem cells (CMG); and the group treated with Cis+ mesenchymal stem cells + beetroot extract (CMBG).

**Table 5:** Changes in means of malondialdehyde (MDA), total antioxidant capacity (TAC), and glutathione (GSH) levels (m. mol/L) in the different study groups.

Group	MDA (m. mol/L)	TAC (m. mol/L)	Glutathione (m. mol/L)
NG	0.08 ± 0.01	44.59 ± 0.22	30.03 ± 1.07 A
CG	0.82 ± 0.04	12.99 ± 0.47	10.63 ± 0.75
CBG	0.62 ± 0.01	30.64 ± 0.50	19.03 ± 0.45
CMG	0.48 ± 0.07	34.9 ± 0.85	23.47 ± 0.55
CMBG	0.22 ± 0.1	40.21 ± 0.38	30.13 ± 0.80 A
LSD	0.041	0.747	1.071

Data are expressed as mean for six-sampled group ± SE. All groups have statistically significant (*p* ≤ 0.05) difference between means, except for groups with the same letter in the same column. The groups are: the normal control group (NG); the Cis group (CG); the group treated with Cis+ beetroot extract (CBG); the group treated with Cis+ mesenchymal stem cells (CMG); and the group treated with Cis+ mesenchymal stem cells + beetroot extract (CMBG). The least significant difference (LSD) values were 0.041, 0.747, and 1.071.

**Table 6:** Expression level of caspase-3, StAR, and nitric oxide synthase (iNOS) genes in response to different treatments.

Group	Caspase-3	StAR	iNOS
NG	1.13 ± 0.01	2.62 ± 0.05 A	1 ± 0.00
CG	4.31 ± 0.02	0.80 ± 0.02	11.6 ± 0.89
CBG	2.85 ± 0.02	1.76 ± 0.06	5.48 ± 0.6 A
CMG	2.21 ± 0.04	2.23 ± 0.03	8.54 ± 0.2
CMBG	1.64 ± 0.03	2.62 ± 0.04 A	3.9 ± 0.44 A
LSD	0.035	0.060	0.742

Data are expressed as mean of gene expression folds in the six-sampled group (compared to GAPDH) ± SE. All groups have statistically significant (*p* ≤ 0.05) difference between means, except for groups with the same letter in the same column. The groups are: the normal control group (NG); the Cis group (CG); the group treated with Cis+ beetroot extract (CBG); the group treated with Cis+ mesenchymal stem cells (CMG); and the group treated with Cis+ mesenchymal stem cells + beetroot extract (CMBG).

## Data Availability

Data sharing not applicable.
